# Encapsulation of anticancer drugs into carbon nanotubes: Heuristic algorithm approach and mathematical model

**DOI:** 10.1371/journal.pone.0321403

**Published:** 2025-05-20

**Authors:** Kanes Sumetpipat, Duangkamon Baowan

**Affiliations:** 1 Department of Mathematics and Computer Science, Kamnoetvidya Science Academy, Rayong, Thailand; 2 Department of Mathematics, Faculty of Science, Mahidol University, Bangkok, Thailand; 3 Centre of Excellence in Mathematics, CHE, Bangkok, Thailand; The HongKong Polytechnic University, HONG KONG

## Abstract

This research combines mathematical derivation and optimization techniques to investigate the non-covalent encapsulation of chemotherapy drugs (fluorouracil, proflavine, methylene blue, and doxorubicin) within carbon nanotubes, aiming to improve targeted drug delivery in cancer therapy. We derive analytical expression for the interaction energy between an atom and an infinite cylinder, and utilize the U-NSGA-III algorithm to optimize the system’s energy by varying molecular positions and tube radius. Optimal tube radii for single- and dual-drug encapsulations are determined. Fixing the tube radius at 10 Å and varying the number of drug molecules, we observe that the shortest distance from the drug’s center of mass to the tube wall is independent of the number of encapsulated molecules, depending only on the drug type. Moreover, equilibrium configurations exhibit two primary patterns, clustering near the tube wall or dispersion around the circumference, suggesting potential control mechanisms for drug release kinetics. This hybrid approach, integrating analytical and computational methods, significantly reduces computational cost, providing a foundation for studying drug-nanocarrier interactions, ultimately accelerating the development of more effective and targeted cancer treatments.

## Introduction

Carbon nanotubes (CNTs) are a family of nano-scaled materials with versatile mechanical, physical, and chemical properties. These properties make them highly applicable in a number of research fields, contributing to advances in environmental protection, food safety, and disease treatment. CNTs show promise as efficient drug delivery systems and in the development of new medical tools and devices. While the literature indicates great applicability of CNTs, further research is needed to fully understand their biocompatibility and standardize their interactions with drugs and biological systems [1].

The development of targeted drug delivery systems is a remarkable area of medical research. These systems aim to precisely deliver therapeutic agents to diseased cells, minimizing systemic toxicity and enhancing treatment efficacy. CNTs, particularly single-walled carbon nanotubes (SWCNTs), have emerged as promising nanocarriers due to their unique properties [2–4]. Their large inner volume, distinct inner and outer surfaces, and cellular penetration capabilities make them ideal candidates for drug delivery. Additionally, SWCNTs have the potential to mitigate the adverse side effects often associated with conventional drug delivery methods [3, 5]. Encapsulating anticancer drugs within CNTs offers several potential advantages.

Mejri *et al*. [6] employ computer simulations to study the stability of cisplatin molecules fit inside the nanotubes and find that the size of the nanotube affects how many drug molecules can fit inside. They also show that the drug molecules are more likely to be released near cancer cells which help to reduce side effects from the drug. Moreover, Dehaghani *et al*. [7] use molecular dynamics simulations to model anticancer drug, Isatin, encapsulated inside a CNT. They find that the Isatin molecule interacts favorably with the inside of the nanotube where eleven Isatin molecules could fit inside a (10, 10) CNT. Furthermore, previous studies have reported the successful encapsulation of other anticancer drugs, such as single molecule of Ifosfamide [8] and that of Lomustine [9], within (10, 10) CNT.

The potential of CNTs for delivering the anticancer drug fluorouracil (5-FU) has been explored by Alexandrovich and Khan [10] using molecular dynamics simulations. Their simulations suggest that a molecule of 5-FU can be effectively encapsulated within the CNT and the specific orientation of the drug molecule within the CNT cavity influences these interactions. Similar research has been carried out by Dehaghani *et al*. [11], where they compare the effectiveness of CNTs and boron nitride nanotubes (BNNTs) for drug delivery. The results show that BNNT is more effective than CNT at encapsulating the drug, this is because the drug interacts more strongly with the BNNT.

Chagovets *et al*. [12] examine the non-covalent interaction of methylene blue dye cation (MB^ + ^) with CNT using molecular dynamics simulation. They discover both parallel and perpendicular orientations of MB^ + ^ on the outer surface of a zigzag (10, 0) and an armchair (6, 6) carbon nanotubes. A weak binding energy between a molecule of methylene blue (MB) and CTNs is theoretically observed by Jauris *et al*. [13] suggesting that the adsorption is physical rather than chemical. Moreover, they find that CNTs with vacancies (missing atoms) have weaker binding energies than those without vacancies, and the binding energy increases as the nanotube diameter grows. Noting that in their study [13], MBs are on the surface of the (5,5) and (8,0) CNTs.

In terms of doxorubicin (DOX), Zhang *et al*. [14] employ a molecular dynamics simulations to investigate how the CNT size and the amount of DOX affect the arrangement of DOX inside the CNT. The results show that DOX molecules prefer to form a single-file helix inside smaller CNTs, whereas in larger CNTs, DOXs form aggregated structures. Further, they report the spacing distance of 3.5–3.7 Å from the DOX and the CNT sidewall. Contreras *et al*. [15] obtain that equilibrium spacing of 3.1–3.9 Å and suggest that encapsulating DOX inside the CNT cavity results in the strongest interaction.

Recent studies have demonstrated the versatility of nanomaterials as drug delivery vehicles. Notably, Chen *et al*. [16] recently show the successful loading of a large peptide drug onto both the interior and exterior of (20, 20) carbon nanotubes (CNTs), highlighting a significant advancement. Beyond CNTs, silicon carbide nanotubes (SiCNTs) [17] and boron nitride nanotubes (BNNTs) [18] have also been successfully utilized as potential drug carriers.

Artificial intelligence (AI) has emerged as a powerful tool in nanotechnology, enabling the optimization of structures and stabilization of systems. In particular, metaheuristic algorithms can be applied directly when objectives and constraints are well-defined. For example, the Unified Non-dominated Sorting Genetic Algorithm III (U-NSGA-III) [19], an extension of NSGA-III [20], is an advanced evolutionary optimization approach particularly efficient in analyzing system stability across various levels of objective complexity. Unlike conventional optimization methods, which may struggle with high-dimensional search spaces and risk getting trapped in local minima, AI-driven techniques in the area of metaheuristic and evolutionary algorithms, like U-NSGA-III, systematically explore a wide range of potential solutions. Through evolutionary processes such as recombination and mutation, the algorithm iteratively refines candidate solutions to achieve better results over successive iterations. This intelligent approach enhances the reliability of stability predictions in nano-scaled systems. Additionally, by using reference directions, U-NSGA-III supports solution diversity and optimizes computational efficiency. Researchers have successfully calculated optimal energy levels and identified novel stable configurations in nano-scaled systems by employing this technique [21–23].

This study investigates the encapsulation of drugs within CNTs using the mathematical derivation and optimization technique. We focus on four well-established drug molecules: fluorouracil (FU, C_4_H_3_FN_2_O_2_) - a frontline treatment for various cancers; proflavine (PF, C_13_H_11_N_3_) - a promising intercalator in cancer therapy and RNA-targeted antiviral treatments; methylene blue (MB, C_16_H_18_ClN_3_S) - an FDA-approved dye and drug used to treat methemoglobinemia; and doxorubicin (DOX, C_27_H_29_NO_11_) - a widely used chemotherapy agent for breast, ovarian, bladder, and lung cancers. The aim is to determine the interaction energy between drugs and CNTs where the van der Waals interaction between an atom on the drug interacting with a cylindrical carbon nanotube is mathematically evaluated through the 6-12 Lennard-Jones potential. Then the optimization method, U-NSGA-III, is employed to assess the stability of the systems.

The energy function and model formation including parameter descriptions and optimization process are detailed in the following section. Numerical results are presented in Sect 2, where the equilibrium configurations of one and two drug molecules encapsulated in CNTs are determined, and optimal tube radii at minima are reported. As the tube radius increases, the arrangement of drugs at stability is described in Sect 2.3. A summary of our work is provided in Sect 3, and calculation details are given in the supporting information.

Highlight of Study’s Contributions:

- **Mathematical Derivation of Interaction Energy:** We provide a detailed mathematical derivation of the van der Waals interaction between drug molecules and CNTs using the 6-12 Lennard-Jones potential, offering a precise analytical approach.
- **Focus on Van der Waals Interactions:** We place a strong emphasis on quantifying the van der Waals interactions, which are critical for understanding the stability of drug-CNT complexes.
- **Application of Genetic Algorithm:** We employ U-NSGA-III to optimize the encapsulation of drug molecules, enabling the identification of stable configurations and optimal tube radii.
- **Comprehensive Study of Four Clinically Relevant Drugs:** Our study investigates the encapsulation of four important drugs: fluorouracil (FU), proflavine (PF), methylene blue (MB), and doxorubicin (DOX), providing a broad analysis of CNT-based drug delivery potential.
- **Analysis of Equilibrium Configurations:** We determine the equilibrium configurations of one and two drug molecules encapsulated in CNTs, revealing the optimal arrangements and spacing distances.
- **Investigation of Tube Radius Influence:** We explore the impact of CNT radius on drug molecule arrangement, describing how drug configurations evolve with increasing tube dimensions.

## 1 Interaction energy and model formation

We employ the Lennard-Jones potential function to calculate the molecular interatomic energy for two non-bonded atoms which can be written as

$$\Phi =-\frac{A}{\rho^{6}}+\frac{B}{\rho^{12}}=\epsilon \left[-2{\left(\frac{\sigma }{\rho }\right)}^{6}+{\left(\frac{\sigma }{\rho }\right)}^{12}\right],$$ (1)

where ρ denotes the distance between two typical points, and $A=2\epsilon \sigma^{6}$ and $B=\epsilon \sigma^{12}$ are attractive and repulsive Lennard-Jones constants, respectively. Further, ϵ denotes a well depth and σ is the van der Waals diameter. Moreover, the mixing rule is utilized in the system of two atomic species which are $\epsilon_{ij}=\sqrt{\epsilon_{i}\epsilon_{j}}$ and $\sigma_{ij}=(\sigma_{i}\;+\;\sigma_{j})/2$. The Lennard-Jones parameters for atoms used in this study are taken from the work of Rappe *et al*. [24].

### 1.1 Energy between atom and cylinder

For two non-bonded molecular structures, the interaction energy can be evaluated using either a discrete atom-atom formulation or by a continuous approach. In the interest of modeling irregularly shaped molecules, such as drugs, an alternative hybrid discrete-continuous approximation can also be used which is given by


$$E^{tot}=\eta \sum_{i}\int \Phi \left(\rho_{i}\right)dS,$$

where η is the surface density of atoms on the molecule which is considered continuous, $\rho_{i}$ is the distance between a typical surface element *dS* on the continuously modeled molecule and atom *i* in the molecule which is modeled as discrete. The total energy is obtained by discretely summing over all atoms in the drug.

The continuous approach is assumed that the atoms at discrete locations on the molecule are averaged over a surface and the molecular interatomic energy is obtained by calculating integrals over the surface of the molecule. On using the Lennard-Jones function defined by (1), the interaction energy between a point (single atom) and a carrier is given by


$$E_{p}=\eta \int_{S}\left(-\frac{A}{\rho^{6}}+\frac{B}{\rho^{12}}\right)dS.$$

For convenience, we define $I_{n}=\int_{S}\frac{1}{\rho^{2n}}dS,$ for n=3,6, and $E_{p}=\eta (-AI_{3}+BI_{6})$.

Now we consider the interaction of an arbitrary point with a cylindrical surface of radius *a* and of infinite length. The cylinder is given parametrically by (acosθ,asinθ,z), where −π<θ<π and −∞<z<∞. Due to the rotational and translational symmetry of the problem, we assume that the point is given in Cartesian coordinates by (δ,0,0). Therefore the distance from the point to a typical surface element of the cylinder is given by


$$\rho^{2}=(a\cos\theta -\delta {)}^{2}+(a\sin\theta {)}^{2}+z^{2}=(a-\delta {)}^{2}+4\delta a\sin^{2}(\theta /2)+z^{2}.$$

Then the integral *I*_*n*_ becomes


$$I_{n}=a\int_{-\infty }^{\infty }\int_{-\pi }^{\pi }\frac{1}{[(a-\delta {)}^{2}+4\delta a\sin^{2}(\theta /2)+z^{2}{]}^{n}}d\theta dz.$$

On using the substitution and changing the limit of integration (see [25] for the integration details), we may deduce


$$I_{n}=\frac{2\pi a}{(a-\delta {)}^{2n-1}}B\left(n-\frac{1}{2},\frac{1}{2}\right)F\left(n-\frac{1}{2},\frac{1}{2};1,-\frac{4a\delta }{(a-\delta {)}^{2}}\right),$$

where *B*(*m*, *n*) is the beta function and F(a,b;c,z) is the hypergeometric function. Hence, the interaction energy between a point and the infinite cylinder is given by


$$E_{p}=\eta \left(-AI_{3}+BI_{6}\right),$$

where η is the mean atomic surface density of CNT which is taken from the mean atomic surface density of the graphene sheet and it is given by $0.3812\;\text{atom}/{\text{\textbackslash{}AA}}^{2}$.

### 1.2 Parameter descriptions

**Fig 1:**
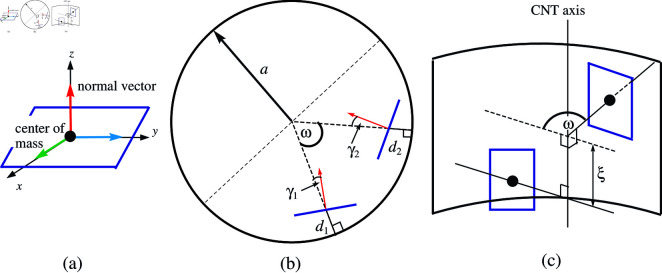
Model formations and parameters for (a) center of mass and normal vector of drug, (b) top-view through CNT axis, and (c) side-view of CNT.

The molecular conformations with the three vectors for fluorouracil, proflavine and methylene blue are as described in [23] and those of the doxorubicin are defined in [22]. Here, we mainly focus on the normal vector as shown in Fig 1(a) to understand the molecular positioning with respect to the CNT.

We define *d*_*i*_ as the shortest distance from the center of mass of the drug *i* to the tube wall. The separation angle between two drug molecules ω is measured from the tube axis, and $\gamma_{i}$ is denoted as the tilt angle of drug *i* in the radial direction of the tube. These three parameters are illustrated in Fig 1(b). Further, ξ is defined as the distance between the two centers of masses along the CNT’s axis as shown in Fig 1(c).

### 1.3 Optimization process and workflow

The methodology in this study largely builds on the optimization framework established by Sumetpipat *et al*. [23], with the addition of doxorubicin to the molecular set. Coordinates for fluorouracil, proflavine, methylene blue, and doxorubicin molecules are obtained from the National Institutes of Health (NIH) PubChem database with CIDs 3385, 7099, 6099, and 31703, respectively [26].

In this setup, all molecules are treated as rigid bodies, neglecting internal bending and twisting. The default position for each molecule is set at the origin (0,0,0). To explore diverse spatial configurations, we introduce five degrees of freedom per molecule: three translational coordinates (*x*, *y*, *z*) and two rotational angles $\left(\theta_{x},\theta_{y}\right)$ around the *x*- and *y*-axes. For a system of *n* molecules, this results in 5*n* decision variables. During optimization process, the spatial coordinates (*x*,*y*,*z*) are constrained to a range of –16 to 16 Å, while the rotational angles are limited to −π to π. The CNT is modeled as an ideal, infinite straight structure aligned with the *z*-axis.

We employ U-NSGA-III from the pymoo library [27], as previously implemented by Sumetpipat *et al*. [23]. This algorithm is particularly effective for single-objective optimization problems in molecular systems. Key parameters, including population size, offspring count, and generation count, are consistently set across multi-molecule configurations. To ensure robustness and reproducibility, each optimization setup is executed 4–10 times with varying seed values. Optimal energy values and molecular configurations are compared and categorized across runs to identify distinct stable structures.

The U-NSGA-III optimization process:

1. **Initialization**: Generate an initial population of candidate solutions. Each solution represents a molecular configuration, defined by five decision variables per molecule: three spatial coordinates (*x*,*y*,*z*) and two rotational angles.
2. **Offspring Generation**: Apply recombination (crossover) and mutation operators to produce new candidate configurations.
3. **Population Combination**: Merge the original set of candidates with the newly generated ones to form a combined pool of solutions.
4. **Non-Dominated Sorting (Single-Objective Version)**: Rank all candidates by sorting them based on their energy values, with the candidates having the lowest energy indicating the most favorable configurations.
5. **Candidate Selection**: Select candidates to form the next generation by choosing those with the lowest energy values. If necessary, select the top candidates to meet the desired population size.
6. **Normalization and Scaling**: Normalize the energy values across candidates to ensure that all energy values fall within a comparable range. This step helps in maintaining numerical stability during the optimization process.
7. **Selection with Diversity**: From the pool of candidates, select the required number of candidates, prioritizing those with the lowest energy values while ensuring diversity in the population. This can be done by considering variations in the spatial coordinates and rotational angles of the candidates, preventing too many similar configurations from being selected.
8. **Termination**: Repeat the following steps (Step 2 to Step 7)—generating offspring, combining populations, sorting by energy, and selecting candidates—until a predetermined number of generations is reached or the energy values stabilize. Finally, output the best configurations with the lowest energy values.

The workflow proceeds as follows:

1. **Formulate and Optimize**: A mathematical system is constructed for each combination of one or two drug molecules with a CNT of fixed radius. Optimization is performed, and the results are analyzed for 10–15 different CNT radii using cubic spline interpolation. These findings are presented in Sects 2.1 and 2.2.
2. **Identify Optimal Radii**: Based on the results from Step 1, optimal CNT radii that minimize energy for each molecule type are identified. The mathematical system is reformulated with these optimal radii, and optimization is performed to obtain precise molecular configurations and energy values. These results are summarized in Tables 1 and 2 of Sects 2.1 and 2.2, respectively.
3. **Expand Optimization for Fixed Radius**: A fixed CNT radius of 10 Å is used to formulate the system for one to six fluorouracil, proflavine and methylene blue molecules, and for one to four doxorubicin molecules. Optimization is performed to identify equilibrium configurations and the results are given in Sect 2.3.

**Table 1:** Minimum energy $E_{min}$ (kcal/mol) obtained from cubic spline interpolation for drugs encapsulated in CNT of radius *a* (Å) where $a_{0}$ (Å) denoted tube radius at zero energy.

Drug	No. of molecules	Zero energy	Global minimum		Local minimum	
$a_{0}$	*a*	$E_{min}$	*a*	$E_{min}$
FU	1	4.5217	5.0611	–26.9849	-	-
	2	4.5199	5.1391	–56.7366	5.5009	–55.6071
PF	1	4.7115	5.2462	–55.8709	-	-
	2	4.7100	5.7604	–125.4071	5.2054	–113.2891
MB	1	4.9791	5.4761	–68.7500	-	-
	2	4.9781	6.0795	–150.4356	5.4805	–139.8456
DOX	1	7.1146	8.1323	–89.4335	-	-
	2	7.1210	8.1662	–187.2074	8.9970	–177.2589

## 2 Numerical results

We determine numerically key parameters as prescribed in Sect 1.2 to characterize molecular alignments. The results of numerical experiments regarding the interaction energy between drug molecules and nanocarriers are presented and interpreted. Assuming a uniform distribution of carbon atoms across the cylinder surface, this analysis focuses solely on the tube radius, neglecting the chirality of CNTs. Additionally, the Python package xyz2graph [28] is utilized to create informative visualizations of the molecular structures, aiding in the analysis and interpretation of results.

### 2.1 Equilibrium configurations of one drug in CNT

We begin by considering the equilibrium configurations of one drug molecule encapsulated in CNT where the energy is minimized. The cubic spline interpolation is utilized to determine the relation of the tube radius giving rise to the minimum energy (see S1 Fig in S1 File). Then the optimum carbon nanotube radius *a* for any drug type can be obtained and they are given in Table 1. The configurations of one drug molecule encapsulated in a CNT at the minimum location are illustrated in supporting information.

The tube radius at zero energy *a*_0_ represents the minimum size required for a CNT to encapsulate the drug. It is worth noting that our finding aligns with Alexandrovich and Khan [10]. They suggest that fluorouracil can be encapsulated in a (7,7) CNT with a radius of approximately 4.5 Å. In our study, the smallest CNT radius required to encapsulate a molecule of fluorouracil is *a*_0_ = 4.5217 Å. In terms of methylene blue, Chagovets *et al*. [12] report that methylene blue can adsorb onto the outer surface of zigzag (10,0) and armchair (6,6) CNTs with radii of 3.915 Å and 4.069 Å, respectively. In contrast, our study indicates that a significantly larger CNT radius, *a*_0_ = 4.980 Å, is required to encapsulate a single methylene blue molecule within the nanotube. This discrepancy in the required CNT radius may explain the observed non-encapsulation behavior in their study.

Moreover, other anticancer drugs such as a single molecule of Ifosfamide [8] and that of Lomustine [9] have been demonstrated the successful encapsulation within a (10,10) CNT with a radius of approximately 6.728 Å. This finding aligns well with our results, further supporting the potential of CNTs as promising nanocarriers for drug delivery.

### 2.2 Equilibrium configurations of two drugs in CNT

**Fig 2:**
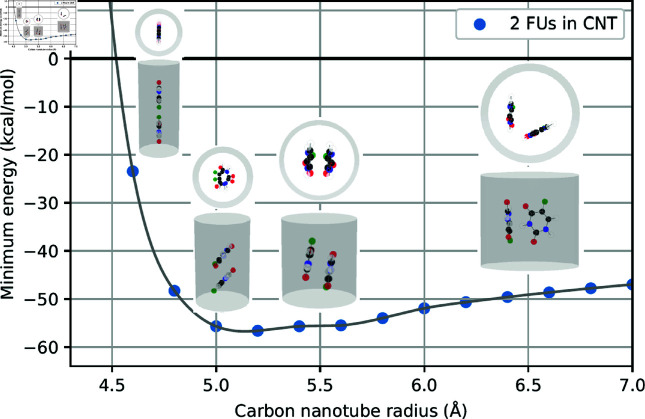
Relation of tube radii at minimum energy and corresponding configurations of two FUs encapsulated in CNTs.

**Fig 3:**
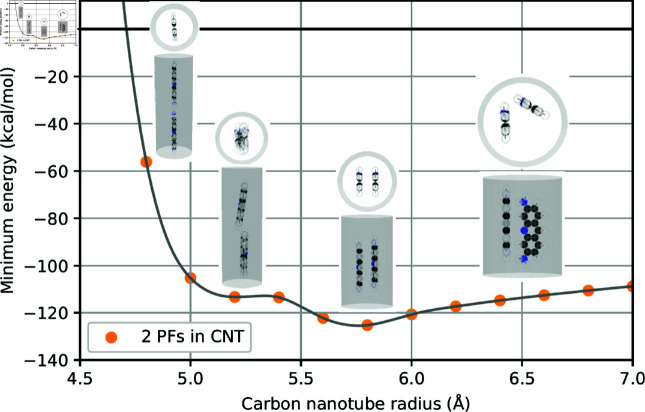
Relation of tube radii at minimum energy and corresponding configurations of two PFs encapsulated in CNTs.

**Fig 4:**
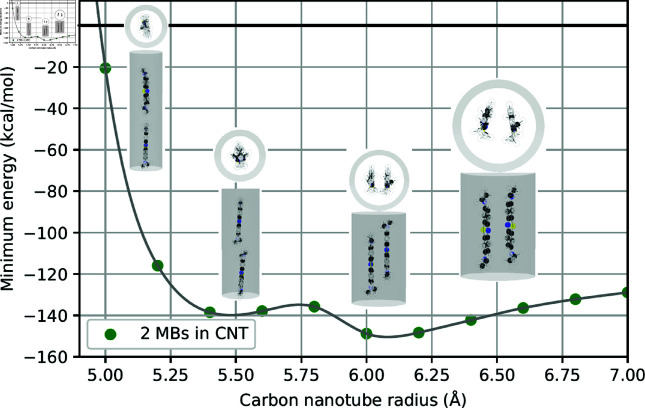
Relation of tube radii at minimum energy and corresponding configurations of two MBs encapsulated in CNTs.

**Fig 5:**
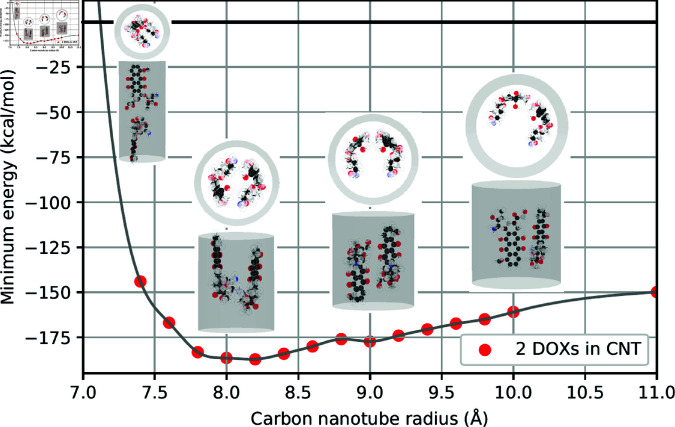
Relation of tube radii at minimum energy and corresponding configurations of two DOXs encapsulated in CNTs.

As observed in Table 1, our results suggest that the doubling of the number of atoms in the system of two drug molecules leads to a corresponding doubling of the energy values at the minima. Here, we graphically illustrate the relation between minimum energy and the tube radius where two minima are observed for fluorouracils, proflavines, methylene blues, and doxorubicins in Figs 2–5, respectively. Note that there will be one minimum energy value (global minimum) for one drug encapsulated in the tube whereas there are two minima (global and local minima) for two drugs encapsulated in the tube. Furthermore, the equilibrium configurations are depicted at the radii corresponding to zero energy, global minimum energy, local minimum energy, and a radius larger than those minima.

In small tube radii *a*_0_, molecules are found to be aligned along the tube axis. As the tube radius increases, molecules either slant away from the axis, although they still remain predominantly aligned, or move further from the tube axis and assume a parallel, symmetric arrangement. In the case of large tube radii, molecules migrate closer to the tube wall, arranging themselves in a side-by-side configuration with a certain separation angle. The configurations of two drug molecules within CNTs are presented in supporting information.

To elucidate the precise equilibrium geometry of the encapsulated drugs, parameters defined in Sect 1.2 are numerical determined and presented in Table 2. Since the two drug molecules are located very close to the tube axis, the tilt angle $\gamma_{i}$ is negligible and therefore omitted from the analysis. For each type of drug, we find that the distances *d*_1_ and *d*_2_ of both molecules are nearly identical. We anticipate that this parameter is unique and crucial for characterizing different drug molecules encapsulated within CNTs. The separation angle between the two molecules at the global minimum is consistently found to be approximately $180^{\circ }$, indicating a parallel arrangement relative to the tube axis.

**Table 2:** Numerical values of parameters describing configurations of two drugs encapsulated in CNTs of radii *a* (Å), where $d_{i}$ (Å) denotes the shortest distance from the drug’s center of mass to the tube surface, ω (∘) is the separation angle between two drugs, and ξ (Å) is the distance between the two centers of masses along the CNT’s axis. Both configurations at global and local minima are analyzed.

Drug	*a*	$d_{1}$	$d_{2}$	ω	ξ
2FUs - Global	5.1391	4.9555	4.9499	178.53	4.8892
2FUs - Local	5.5009	4.0069	4.0080	179.99	2.2454
2PFs - Global	5.7604	4.0273	4.0271	165.50	0.7731
2PFs - Local	5.2054	5.0112	4.9978	171.15	12.0428
2MBs - Global	6.0795	4.2997	4.2997	171.11	3.2124
2MBs - Local	5.4805	5.0498	5.1245	3.10	15.4377
2DOXs - Global	8.1662	4.7572	4.7540	175.47	2.4735
2DOXs - Local	8.9970	4.6268	4.6269	115.34	1.8943

However, in the case of 2MBs-Local, the angle of separation ω is determined to be $3.10^{\circ }$. This value is observed in numerical simulations as the two methylene blue molecules are positioned near the center of the CNT, leading to molecular accumulation in the same direction with a slight offset from the center.

### 2.3 Encapsulation of drugs in CNT of radius 10 Å

We further investigate the equilibrium configurations of drugs encapsulated in a large cylindrical container where the CNT radius is assumed to be *a* = 10 Å. A number of drug molecules are assumed to be one to six to examine both the interaction energy between drugs and containers, and that among drug molecules. We find that all four types of drug molecules studied here are prefer to move closer to the tube wall when the tube cavity is getting larger.

**Fig 6:**
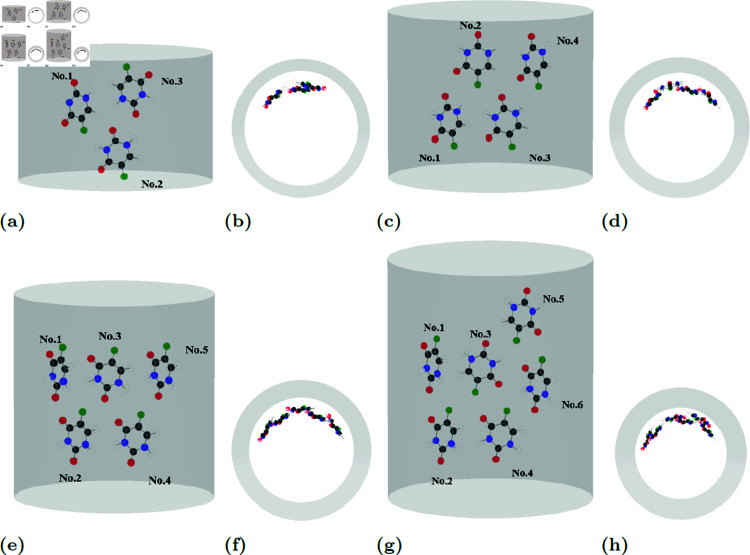
FUs maintain 3.47 Å distance from CNT wall, arranging in trellis and triangular patterns. Examples of equilibrium locations both side- and top-view of FUs (a), (b) three, (c), (d) four, (e), (f) five and (g), (h) six molecules in CNTs of radii 10 Å.

**Fig 7:**
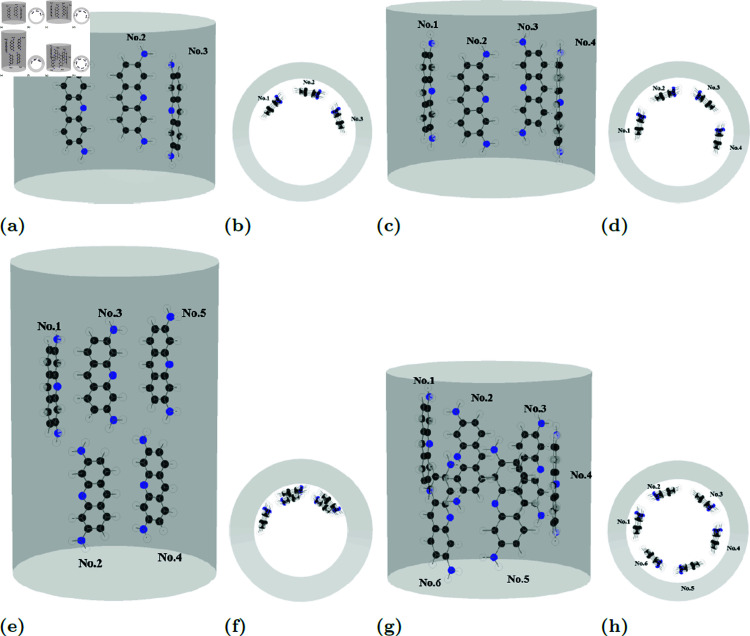
Planar structure of PF results in 3.50 Å spacing from CNT wall, and arranging in two-layer clusters or spiral staircase patterns with varied orientations. Examples of equilibrium locations both side- and top-view of PFs (a), (b) three, (c), (d) four, (e), (f) five and (g), (h) six molecules in CNTs of radii 10 Å.

Numerical values for parameters characterizing the molecular encapsulation configurations are presented in Tables 3–6. These values are determined at equilibrium, where the optimization process converged to a minimum energy state. At equilibrium, drug molecules exhibit two primary spatial arrangements, either clustering near the tube wall (Figs 6(g), 6(h)) or dispersing around the tube circumference in proximity to the wall (Figs 7(g), 7(h)).

In the case of fluorouracil, the calculated distance between fluorouracil and the tube wall *d*_*i*_ is approximately 3.47 Å. This value closely aligns with the inter-layer distance of carbon graphene sheets, likely due to the predominance of a planar carbon ring in the fluorouracil structure. The tilt angle $\gamma_{i}$ is nearly zero indicating that the drug’s normal vector points towards the tube center and the drugs are parallel to the tube wall. Moreover, multiple fluorouracils tend to arrange in a trellis-like pattern along the CNT wall as confirmed by the values of $\xi_{ij}$ and illustrated in Fig 6 for three to six fluorouracils. We also observe an equilateral triangle formation of any three molecules of fluorouracils near the tube wall which is in an agreement with the pervious work of authors for the absorption of fluorouracils on the graphene and that on the C_60_ fullerene [23]. The numerical values of parameters characterizing equilibrium configuration of fluorouracils are given in Table 3.

**Table 3:** Parameters values defined in Sect 1.2 of fluorouracil encapsulated in CNT of radius a=10 Å with minimum energy $E_{min}$ (kcal/mol).

Molecules	1	2	3	4	5	6
*E*_*min*_	–19.5143	–40.4632	–61.6527	–82.9085	–103.5628	–125.8061
*d*_1_	3.4707	3.4751	3.4745	3.4708	3.4666	3.4874
*d*_2_	-	3.4729	3.4737	3.4722	3.4674	3.4608
*d*_3_	-	-	3.4746	3.4914	3.4633	3.4816
*d*_4_	-	-	-	3.4757	3.4794	3.4795
*d*_5_	-	-	-	-	3.4883	3.4918
*d*_6_	-	-	-	-	-	3.4622
$\gamma_{1}$	1.79	2.22	1.78	1.83	1.87	0.39
$\gamma_{2}$	-	2.38	1.25	1.33	1.56	0.80
$\gamma_{3}$	-	-	1.03	2.68	0.86	1.20
$\gamma_{4}$	-	-	-	0.38	0.62	0.42
$\gamma_{5}$	-	-	-	-	1.51	0.97
$\gamma_{6}$	-	-	-	-	-	1.65
$\xi_{12}$	-	0.6721	5.3106	6.5147	6.8833	5.9931
$\xi_{23}$	-	-	7.5939	6.7966	6.7095	7.1688
$\xi_{34}$	-	-	-	6.6071	7.0331	7.1844
$\xi_{45}$	-	-	-	-	7.3328	7.1071
$\xi_{56}$	-	-	-	-	-	0.5451
$\omega_{12}$	-	53.55	41.39	30.09	25.96	35.14
$\omega_{23}$	-	-	17.04	28.26	32.27	24.90
$\omega_{34}$	-	-	-	28.99	25.91	32.02
$\omega_{45}$	-	-	-	-	30.75	25.75
$\omega_{56}$	-	-	-	-	-	54.19

**Table 4:** Parameters values defined in Sect 1.2 of proflavines encapsulated in CNT of radius a=10 Å with minimum energy $E_{min}$ (kcal/mol).

Molecules	1	2	3	4	5	6
*E*_*min*_	–38.6606	–93.5247	–142.0661	–190.6414	–238.5398	–289.9497
*d*_1_	3.5032	3.5042	3.5042	3.5047	3.5046	3.5160
*d*_2_	-	3.5041	3.5053	3.5058	3.5054	3.5065
*d*_3_	-	-	3.5043	3.5057	3.5053	3.5184
*d*_4_	-	-	-	3.5045	3.5069	3.5229
*d*_5_	-	-	-	-	3.5049	3.5137
*d*_6_	-	-	-	-	-	3.5292
$\gamma_{1}$	2.11	2.48	2.45	2.53	2.55	2.72
$\gamma_{2}$	-	2.47	2.13	2.08	1.74	2.59
$\gamma_{3}$	-	-	2.48	2.22	2.16	2.45
$\gamma_{4}$	-	-	-	2.48	2.51	1.65
$\gamma_{5}$	-	-	-	-	1.62	2.09
$\gamma_{6}$	-	-	-	-	-	3.32
$\xi_{12}$	-	1.2844	1.1310	1.1142	11.6657	1.3529
$\xi_{23}$	-	-	1.2931	1.2971	12.7901	1.3065
$\xi_{34}$	-	-	-	1.1172	11.6549	1.2810
$\xi_{45}$	-	-	-	-	12.7723	1.3840
$\xi_{56}$	-	-	-	-	-	1.3324
$\omega_{12}$	-	55.85	59.80	59.83	49.90	56.07
$\omega_{23}$	-	-	55.88	55.93	9.83	64.84
$\omega_{34}$	-	-	-	59.83	49.90	56.52
$\omega_{45}$	-	-	-	-	9.85	64.80
$\omega_{56}$	-	-	-	-	-	56.53

Proflavines molecules, with their planar three-ring structure, exhibit a preferred orientation parallel to the tube wall, minimizing the distance *d*_*i*_ between the molecule and the CNT wall to approximately 3.50 Å. This value closely aligns with the interlayer spacing in graphene. As illustrated in Fig 7, the tilt angle $\gamma_{i}$ tends toward zero, confirming the parallel orientation.

Two distinct molecular alignments are observed for proflavines molecules within the CNT. For five proflavines molecules, a two-layer cluster forms near the wall, characterized by alternating separation angles $\omega_{ij}$ and a layer shift $\xi_{ij}$ of approximately 12 Å. Note that the length of a proflavines molecule is approximately 11.3867 Å. In other cases as shown in Fig 7, a linear arrangement prevails, resulting in a side-by-side pattern with a separation angle $\omega_{ij}$ of around $60^{\circ }$ and a layer shift $\xi_{ij}$ of approximately 1.3 Å. This arrangement resembles a spiral staircase.

The structure of methylene blue is more sophisticated with more branches of atoms, demand more spatial accommodation within CNTs compared to the simpler fluorouracil and proflavine molecules. This increased spatial requirement is reflected in the calculated distances *d*_*i*_ between the methylene blue center of mass and the tube wall, which range from 3.9 to 4.0 Å. Additionally, methylene blue molecules exhibit larger tilt angel $\gamma_{i}$ within the CNTs.

**Fig 8:**
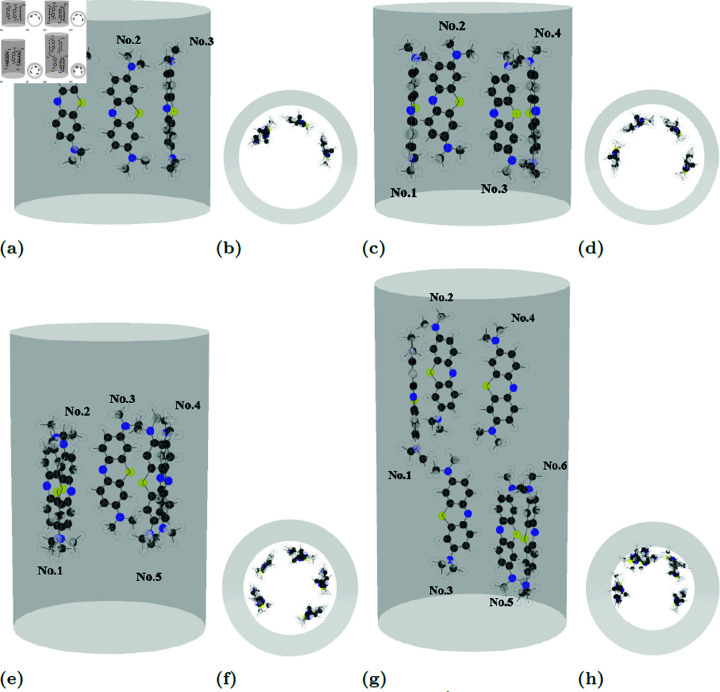
Due to its larger size, MB exhibits 3.9–4.0 Å spacing from CNT wall and larger tilt angles. It forms linear, $60^{\circ }$ spaced arrangements, but shifts to a two-layer structure with six molecules due to spatial constraints. Examples of equilibrium locations both side- and top-view of MBs (a), (b) three, (c), (d) four, (e), (f) five and (g), (h) six molecules in CNTs of radii 10 Å.

Multiple methylene blue molecules tend to adopt a linear arrangement along the CNT wall, characterized by small offset values $\xi_{ij}$ and separation angles $\omega_{ij}$ of approximately $60^{\circ }$. This side-by-side configuration is observed for systems containing one to five methylene blue molecules. However, for six methylene blue molecules, the limited circumferential space within the CNT requires a two-layer arrangement, resulting in larger offset values and smaller separation angles. Visual representations of these configurations are provided in Fig 8, and the corresponding numerical data are tabulated in Table 5.

**Table 5:** Parameters values defined in Sect 1.2 of methylene blue encapsulated in CNT of radius a=10 Å with minimum energy $E_{min}$ (kcal/mol).

Molecules	1	2	3	4	5	6
*E*_*min*_	–50.3980	–106.9736	–162.5101	–218.1929	–272.7699	–324.3523
*d*_1_	3.9123	3.9108	3.9272	3.9193	3.9218	3.9257
*d*_2_	-	3.9125	3.9246	3.9389	3.9446	3.9946
*d*_3_	-	-	3.9023	3.9040	3.9413	3.9367
*d*_4_	-	-	-	3.9257	3.9779	3.9330
*d*_5_	-	-	-	-	3.9840	3.9296
*d*_6_	-	-	-	-	-	3.9194
$\gamma_{1}$	2.66	3.69	9.02	5.20	5.67	9.18
$\gamma_{2}$	-	9.04	5.70	5.50	10.34	11.89
$\gamma_{3}$	-	-	2.81	1.74	9.38	9.00
$\gamma_{4}$	-	-	-	4.53	10.74	6.30
$\gamma_{5}$	-	-	-	-	11.83	5.73
$\gamma_{6}$	-	-	-	-	-	9.03
$\xi_{12}$	-	0.2030	1.1557	0.5752	1.0481	0.7274
$\xi_{23}$	-	-	0.4518	1.3186	1.9794	16.2379
$\xi_{34}$	-	-	-	0.4778	0.5901	14.4833
$\xi_{45}$	-	-	-	-	0.3292	15.9611
$\xi_{56}$	-	-	-	-	-	2.8442
$\omega_{12}$	-	62.31	62.93	63.76	63.20	62.12
$\omega_{23}$	-	-	64.12	63.20	63.87	14.97
$\omega_{34}$	-	-	-	64.38	62.50	48.70
$\omega_{45}$	-	-	-	-	62.22	14.71
$\omega_{56}$	-	-	-	-	-	66.26

Due to the large size and branching structure of doxorubicin molecules, a CNT with a radius of 10 Å is relatively small to accommodate multiple encapsulated doxorubicins. Consequently, this study focuses on configurations with one to four doxorubicin molecules. The shortest distance between the center of mass of each drug molecule and the tube wall *d*_*i*_ remains relatively constant, ranging from 4.6 to 4.9 Å. However, the non-planar structure of doxorubicin results in larger tilt angles $\gamma_{i}$. The trends observed in the intermolecular interaction parameters, $\xi_{ij}$ and $\omega_{ij}$, are less intuitive.

**Fig 9:**
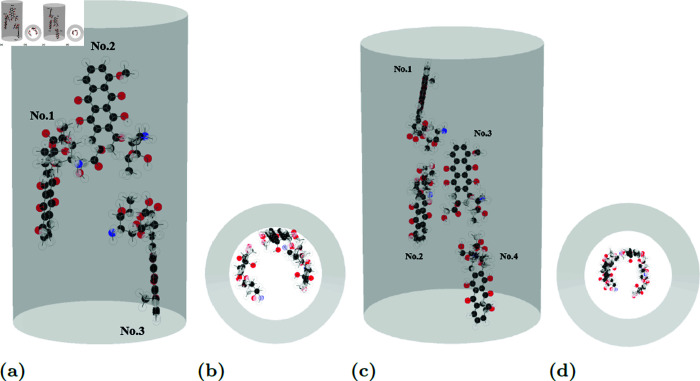
Due to its large size, a 10 Å CNT accommodates only 1–4 molecules. DOX maintains a 4.6–4.9 Å center-of-mass distance from CNT wall, with larger tilt angles. Examples of equilibrium locations both side- and top-view of DOXs (a), (b) three and (c), (d) four molecules in CNTs of radii 10 Å.

We observe that the doxorubicin molecules arrange themselves within the CNT in a manner consistent with previous findings [14]. In smaller CNTs, the molecules form a single linear chain (Fig 5), while in larger CNTs, they aggregate into a clustered configuration (Fig 9). The distance from the closest atoms on doxorubicin to the tube wall in our study is 2.52 Å. However, the intermolecular distance *d*_*i*_ of doxorubicin molecules in our study is larger than the value of 3.5–3.7 Å reported in [14]. Fig 9 and Table 6 present the equilibrium configurations and corresponding parameter values, respectively.

**Table 6:** Parameters values defined in Sect 1.2 of doxorubicin encapsulated in CNT of radius a=10 Å with minimum energy $E_{min}$ (kcal/mol).

Molecules	1	2	3	4
*E*_*min*_	–78.6837	–161.9740	–241.1098	–304.8341
*d*_1_	4.5786	4.5766	4.5933	4.9081
*d*_2_	-	4.5758	4.5607	4.5692
*d*_3_	-	-	4.5604	4.5775
*d*_4_	-	-	-	4.6109
$\gamma_{1}$	14.73	11.12	10.59	24.29
$\gamma_{2}$	-	11.16	12.93	11.92
$\gamma_{3}$	-	-	13.24	11.06
$\gamma_{4}$	-	-	-	9.52
$\xi_{12}$	-	1.2325	5.9990	15.0943
$\xi_{23}$	-	-	14.4945	1.9867
$\xi_{34}$	-	-	-	13.7845
$\omega_{12}$	-	94.41	99.4739	4.92
$\omega_{23}$	-	-	67.0904	98.39
$\omega_{34}$	-	-	-	100.26

Furthermore, we observe that in a CNT with a radius of 10 Å, fluorouracils, proflavines, and methylene blues exhibit arrangements similar to those found in graphene-based nanocarriers [23]. This similarity can be attributed to the relatively large CNT radius, which minimizes curvature effects, making the CNT surface resemble a nearly flat graphene sheet. Note that a molecule of doxorubicin is too large comparing to the tube used here. The encapsulation of multiple molecules of drugs in a CNT is considered as a larger system for the molecular dynamic simulation, therefore less comparison can be made with our study.

It is important to acknowledge the limitations of our model, specifically the use of a continuous approximation that disregards carbon nanotube chirality and focuses solely on tube radius. While chirality is crucial for electrical properties, our investigation is limited to van der Waals interactions, which dictate the equilibrium configuration of drug encapsulation. Moreover, given the short-range nature of van der Waals forces, the assumption of infinitely long CNTs is expected to affect the overall energy magnitude, but not the equilibrium distances between the encapsulated molecules and the nanotube.

## 3 Summary

This research investigates the combining application of explicit formulas and U-NSGA-III algorithm to predict stable configurations of drug molecules interacting with cylindrical carbon nanotubes. We employ fluorouracil, proflavine, methylene blue, and doxorubicin as model chemotherapy drugs. To accurately represent the irregular shapes of these molecules, a hybrid discrete-continuous approximation based on Lennard-Jones potential is utilized. By deriving analytical expressions for the interaction energy between an atom and an infinite cylinder, we establish a foundation for optimizing the system’s energy using the U-NSGA-III algorithm. This optimization process enables the efficient determination of equilibrium positions for both drug molecules and the nanocarrier.

To begin, we investigate the equilibrium configurations of one and two drug molecules encapsulated within infinite carbon nanotubes, focusing on determining the optimal tube radii for stable states. Note that the tube is assumed to be infinitely long, which does not affect the equilibrium configurations due to the short-range nature of the Lennard-Jones potential. We identify both the minimum tube radius required to accommodate each drug type and the optimal radius that minimizes the system’s energy. When considering two drug molecules, we observe two distinct minimum-energy configurations, one where the molecules align along the tube axis and another where they arrange themselves in a parallel orientation. Numerical results detailing the parameters of these two-molecule configurations are presented in Table 2.

Additionally, we investigate the encapsulation of multiple drug molecules within a 10 Å radius carbon nanotube. We consider one to six molecules of fluorouracil, proflavine, and methylene blue, and one to four molecules of doxorubicin due to its larger size. By analyzing the equilibrium configurations of these systems, we observe a consistent trend, the shortest distance between the drug center of mass and the tube wall remains constant for each drug type, regardless of the number of encapsulated molecules. The arrangement of drugs at equilibrium exhibits two primary patterns which is clustering near the tube wall or dispersing around the circumference while maintaining proximity to the wall. Interestingly, fluorouracil, proflavine, and methylene blue exhibit arrangements similar to those found in graphene-based nanocarriers [23] due to the minimal curvature of the large-radius nanotube, which approximates a flat graphene sheet.

Simulating the encapsulation of multiple drug molecules within a CNT requires a larger system size, which limits direct comparisons with our study. This theoretical study, grounded in mathematical principles and heuristic algorithms, yields results that are highly consistent with those obtained from computationally intensive simulations. This foundational work provides crucial insights into the design of nanocarriers for drug delivery systems.

In general, it can be said that this research holds significant potential for advancing cancer therapy through improved drug delivery. By determining optimal carbon nanotube configurations for encapsulating chemotherapy drugs, our work provides insights into designing more efficient and targeted nanocarriers. The consistent drug-wall distance observed across varying drug loads simplifies predictive modeling for drug release, while the identified clustering and dispersion patterns offer potential control mechanisms for drug release kinetics. Ultimately, our hybrid approach, which significantly reduces computational costs, can accelerate the rapid screening and design of drug-nanocarrier interactions, paving the way for the development of more effective, personalized, and less toxic cancer treatments.

## Supporting information

S1 File Illustrations of equilibrium configurations of drugs encapsulated in carbon nanotubes (PDF)

## References

[1] Brito CL, Silva JV, Gonzaga RV, La-Scalea MA, Giarolla J, Ferreira EI. A review on carbon nanotubes family of nanomaterials and their health field. ACS Omega. 2024;9(8):8687–708. doi: 10.1021/acsomega.3c08824 38434894 PMC10905599

[2] Madani SY, Naderi N, Dissanayake O, Tan A, Seifalian AM. A new era of cancer treatment: carbon nanotubes as drug delivery tools. Int J Nanomedicine. 2011;6:2963–79. doi: 10.2147/IJN.S16923 22162655 PMC3230565

[3] Elhissi A, Ahmed W, Dhanak VR, Subramani K. Chapter 20 - Carbon nanotubes in cancer therapy and drug delivery. In: Subramani K, Ahmed W, editors. Emerging nanotechnologies in dentistry. Micro and nano technologies. Boston: William Andrew Publishing; 2012. p. 347–63.

[4] Saleemi MA, Kong YL, Yong PVC, Wong EH. An overview of recent development in therapeutic drug carrier system using carbon nanotubes. J Drug Deliv Sci Technol. 2020;59:101855. doi: 10.1016/j.jddst.2020.101855

[5] Saeed M, Haq RSU, Ahmed S, Siddiqui F, Yi J. Recent advances in carbon nanotubes, graphene and carbon fibers-based microwave absorbers. J Alloys Compounds. 2024;970:172625. doi: 10.1016/j.jallcom.2023.172625

[6] Mejri A, Vardanega D, Tangour B, Gharbi T, Picaud F. Encapsulation into carbon nanotubes and release of anticancer Cisplatin drug molecule. J Phys Chem B. 2015;119(2):604–11. doi: 10.1021/jp5102384 25514358

[7] Dehaghani MZ, Yousefi F, Seidi F, Bagheri B, Mashhadzadeh AH, Naderi G, et al. Encapsulation of an anticancer drug Isatin inside a host nano-vehicle SWCNT: a molecular dynamics simulation. Sci Rep. 2021;11(1):18753. doi: 10.1038/s41598-021-98222-2 34548596 PMC8455564

[8] Yoosefian M, Sabaei S, Etminan N. Encapsulation efficiency of single-walled carbon nanotube for Ifosfamide anti-cancer drug. Comput Biol Med. 2019;114:103433. doi: 10.1016/j.compbiomed.2019.103433 31514075

[9] Cao M, Wu D, Yoosefian M, Sabaei S, Jahani M. Comprehensive study of the encapsulation of Lomustine anticancer drug into single walled carbon nanotubes (SWCNTs): Solvent effects, molecular conformations, electronic properties and intramolecular hydrogen bond strength. J Molecul Liquids. 2020;320:114285. doi: 10.1016/j.molliq.2020.114285

[10] Alexandrovich TP, Khan A. Molecular insights into the encapsulation of fluorouracil molecule inside the single-walled carbon nanotubes. Diamond Relat Mater. 2022;124:108900. doi: 10.1016/j.diamond.2022.108900

[11] Zarghami Dehaghani M, Yousefi F, Sajadi SM, Tajammal Munir M, Abida O, Habibzadeh S, et al. Theoretical encapsulation of fluorouracil (5-FU) anti-cancer chemotherapy drug into carbon nanotubes (CNT) and boron nitride nanotubes (BNNT). Molecules. 2021;26(16):4920. doi: 10.3390/molecules26164920 34443508 PMC8398462

[12] Chagovets V, Kosevich M, Stepanian S, Boryak O, Shelkovsky V, Orlov V. Noncovalent interaction of methylene blue with carbon nanotubes: theoretical and mass spectrometry characterization. J Phys Chem C. 2012;116(38):20579–90.

[13] Jauris IM, Fagan SB, Adebayo MA, Machado FM. Adsorption of acridine orange and methylene blue synthetic dyes and anthracene on single wall carbon nanotubes: a first principle approach. Comput Theor Chem. 2016;1076:42–50. doi: 10.1016/j.comptc.2015.11.021

[14] Zhang L, Peng G, Li J, Liang L, Kong Z, Wang H, et al. Molecular dynamics study on the configuration and arrangement of doxorubicin in carbon nanotubes. J Molecul Liquids. 2018;262:295–301. doi: 10.1016/j.molliq.2018.04.097

[15] Contreras M, Torres C, Villarroel I, Rozas R. Molecular dynamics assessment of doxorubicin–carbon nanotubes molecular interactions for the design of drug delivery systems. Struct Chem. 2019;30(1):369–84.

[16] Chen Q, Zhou J, Sun R. Carbon nanotube loading strategies for peptide drugs: insights from molecular dynamics simulations. Langmuir. 2024;40(26):13515–26. doi: 10.1021/acs.langmuir.4c00973 38887887

[17] Madadi Mahani N. A density functional theory study on silicon carbide and carbon nanotube (11, 11) as drug delivery of Olutasidenib. Theor Chem Acc. 2024;144(2). doi: 10.1007/s00214-024-03157-2

[18] Krishna AB, Suvilal A, Vamadevan R, Babu JS. Exploring boron nitride nanotubes as potential drug delivery vehicles using density functional theory and molecular dynamics – an overview. J Molecul Liquids. 2024;413:125968. doi: 10.1016/j.molliq.2024.125968

[19] Seada H, Deb K. A unified evolutionary optimization procedure for single, multiple, and many objectives. IEEE Trans Evol Computat. 2016;20(3):358–69. doi: 10.1109/tevc.2015.2459718

[20] Deb K, Jain H. An evolutionary many-objective optimization algorithm using reference-point-based nondominated sorting approach, part i: solving problems with box constraints. IEEE Trans Evol Computat. 2014;18(4):577–601. doi: 10.1109/tevc.2013.2281535

[21] Ganazzoli F, Raffaini G. Classical atomistic simulations of protein adsorption on carbon nanomaterials. Curr Opin Colloid Interface Sci. 2019;41:11–26. doi: 10.1016/j.cocis.2018.11.008

[22] Sumetpipat K, Baowan D. Stable configurations of DOXH interacting with graphene: heuristic algorithm approach using NSGA-II and U-NSGA-III. Nanomaterials. 2022;12(22):4097. 10.3390/nano12224097 PMC9693072 36432383

[23] Sumetpipat K, Baowan D, Tiangtrong P. Mathematical modeling and optimization technique of anticancer antibiotic adsorption onto carbon nanocarriers. Sci Rep. 2024;14(1):11988. doi: 10.1038/s41598-024-62483-4 38796555 PMC11127958

[24] Rappe AK, Casewit CJ, Colwell KS, Goddard WA III, Skiff WM. UFF, a full periodic table force field for molecular mechanics and molecular dynamics simulations. J Am Chem Soc. 1992;114(25):10024–35. doi: 10.1021/ja00051a040

[25] Baowan D, Cox BJ, Hilder TA, Hill JM, Thamwattana N. Modelling and Mechanics of Carbon-based Nanostructured Materials. William Andrew; 2017.

[26] Kim S, Chen J, Cheng T, Gindulyte A, He J, He S, et al. PubChem 2023 update. Nucleic Acids Res. 2023;51(D1):D1373–80. doi: 10.1093/nar/gkac956 36305812 PMC9825602

[27] Blank J, Deb K. Pymoo: multi-objective optimization in python. IEEE Access. 2020;8:89497–509. doi: 10.1109/access.2020.2990567

[28] Zotko M. xyz2graph: molecular structure visualization; 2018. Available from: https://github.com/zotko/xyz2graph

