# Supplementary material for: Encapsulation of anticancer drugs into carbon nanotubes: Heuristic algorithm approach and mathematical model
Source: PLoS One. 2025 May 20;20(5):e0321403. doi: 10.1371/journal.pone.0321403 (PMC12091757; doi:10.1371/journal.pone.0321403)
Supplement: S1 File [file pone.0321403.s001.pdf]

# Supporting Information - Encapsulation of anticancer drugs into carbon nanotubes: Heuristic algorithm approach and mathematical model

December 18, 2024

## 1 Curve fitting for optimum tube radius

For any given carbon nanotube radius, we determine the energy value at the equilibrium, then utilize the cubic spline interpolation for the curve fitting as shown in Fig. S.1. Consequently, the optimum carbon nanotube radius for any drug type is numerically reported in Table 1.

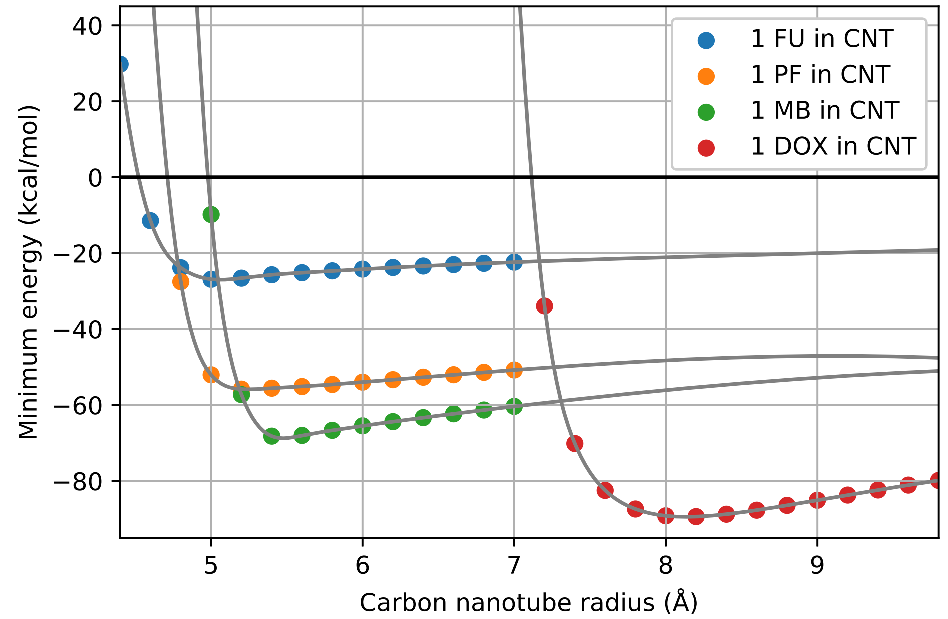


(a) One drug molecule

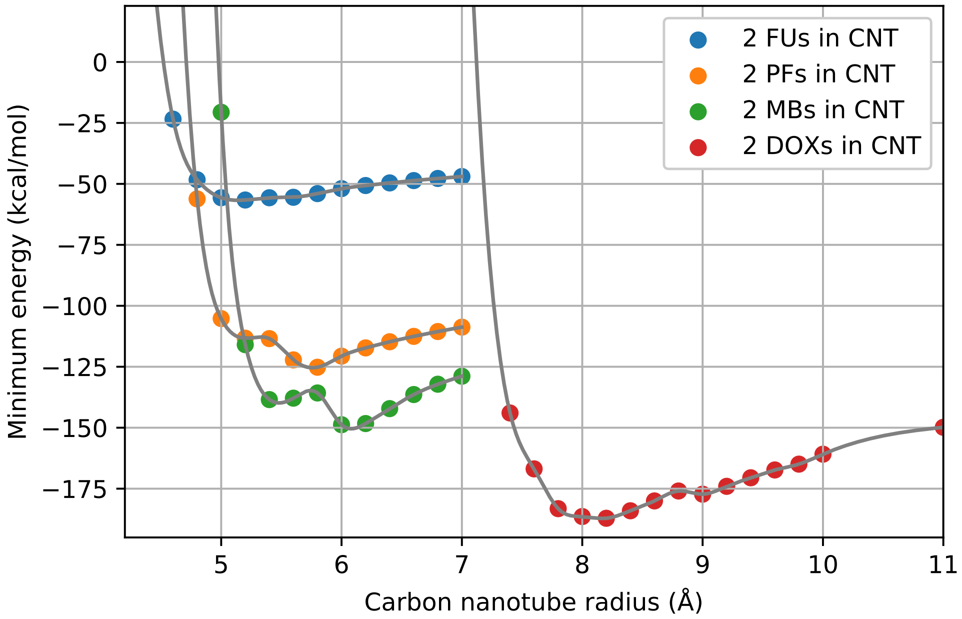


(b) Two drug molecules

Figure S.1: Curve fitting of minimum energy for various radii of carbon nanotube where (a) one and (b) two drug molecules encapsulated inside the tubes.

# 2 Equilibrium configuration of one drug molecule encapsulated in carbon nanotube

Figures S.2 - S.5 represent the equilibrium configurations of one molecule of fluorouracil, proflavine, methylene blue, and doxorubicin, respectively, inside carbon nanotubes. These configurations correspond to the carbon nanotube radii that minimize the energy for each drug type as detailed in Table 1.

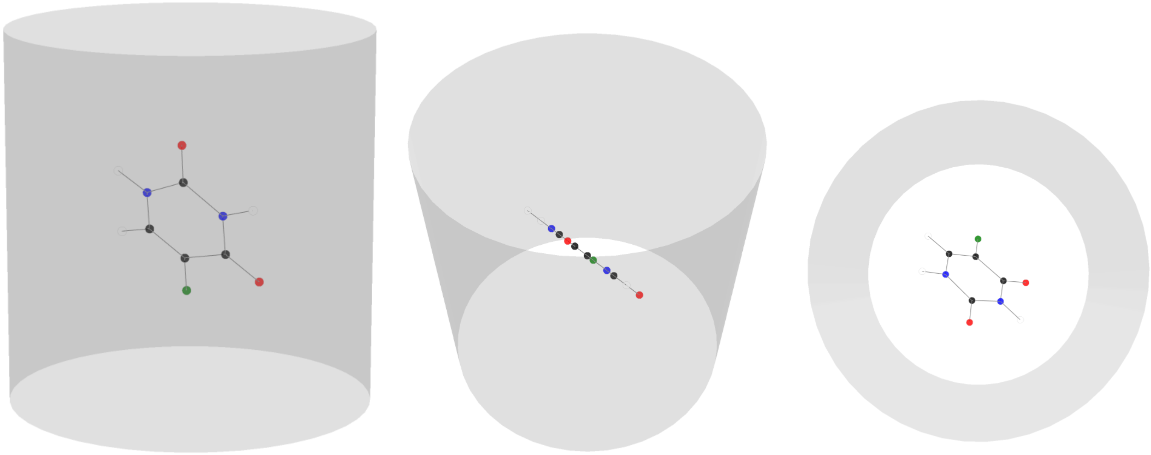

(a) Front view (b) Side view (c) Top view

Figure S.2: One fluorouracil in CNT of radius 5.0611 Å.

Figure S.6 illustrates the relationship between carbon nanotube radii and the possible configurations for encapsulating a single fluorouracil. This information is directly applicable to the case of two fluorouracils encapsulated within a carbon nanotube, as shown in Fig. 2. Consequently, the encapsulation of single molecules like proflavine, methylene blue, or doxorubicin within a carbon nanotube can be understood by referring to Figs. 3, 4 and 5, respectively.

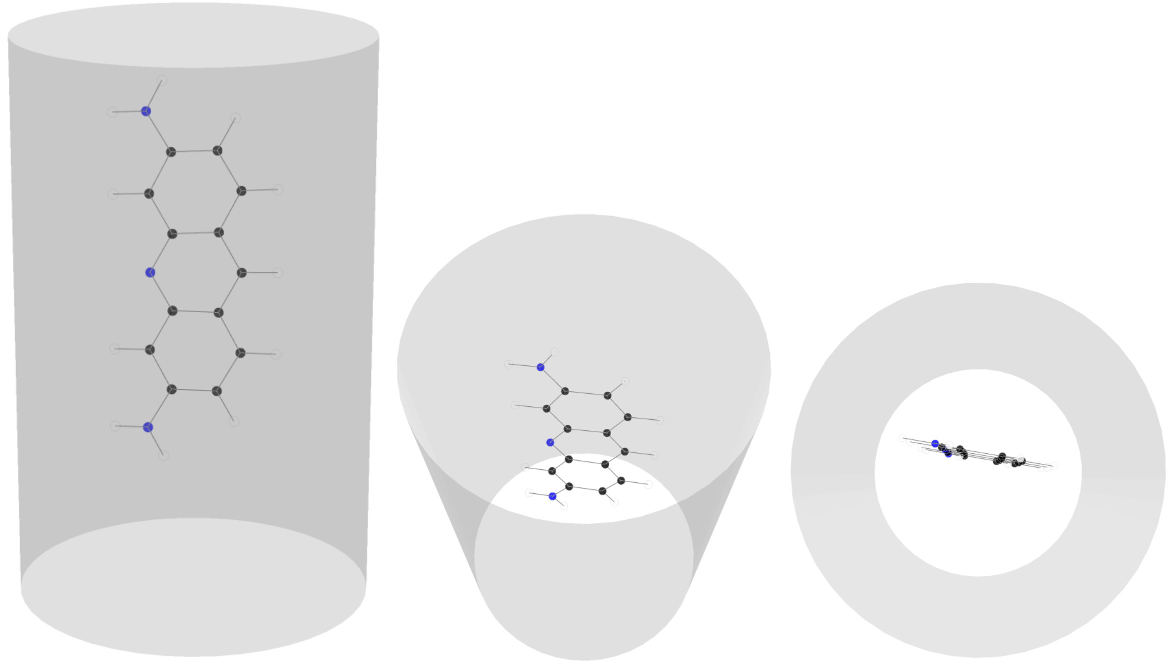

(a) Front view (b) Side view (c) Top view

Figure S.3: One proflavine in CNT of radius 5.2462 Å.

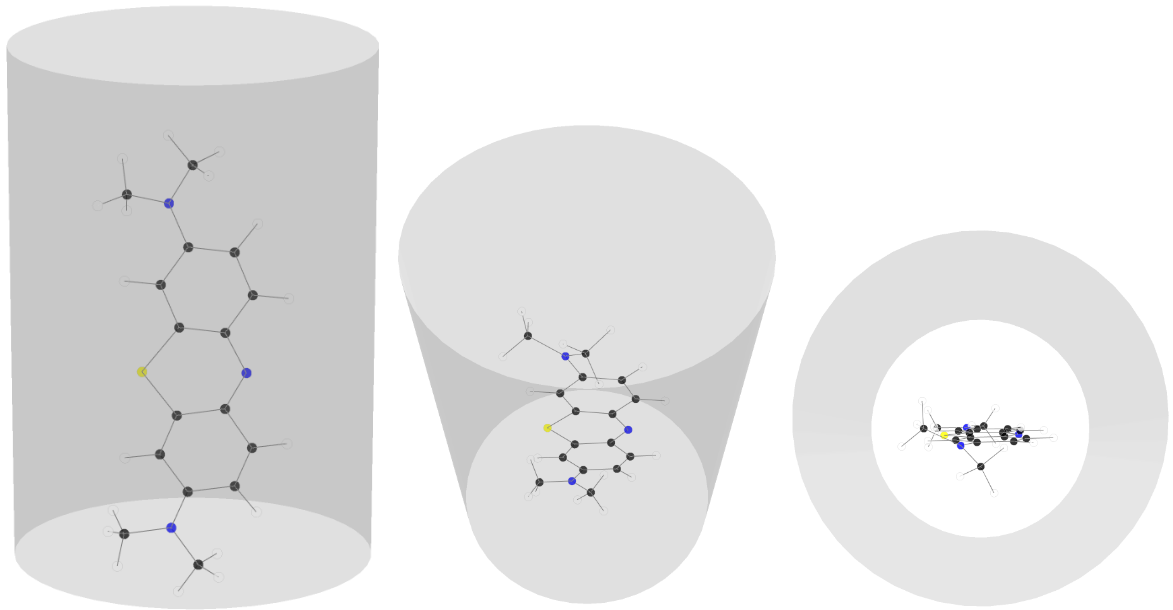

(a) Front view (b) Side view (c) Top view

Figure S.4: One methylene blue in CNT of radius 5.4761 Å.

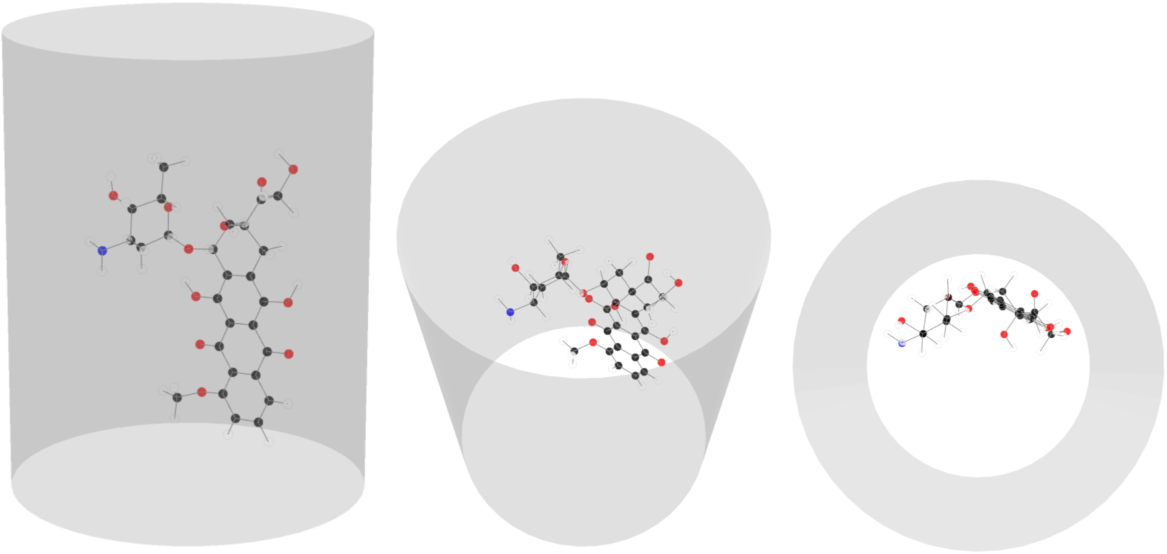

(a) Front view (b) Side view (c) Top view

Figure S.5: One doxorubicin in CNT of radius 8.1323 Å.

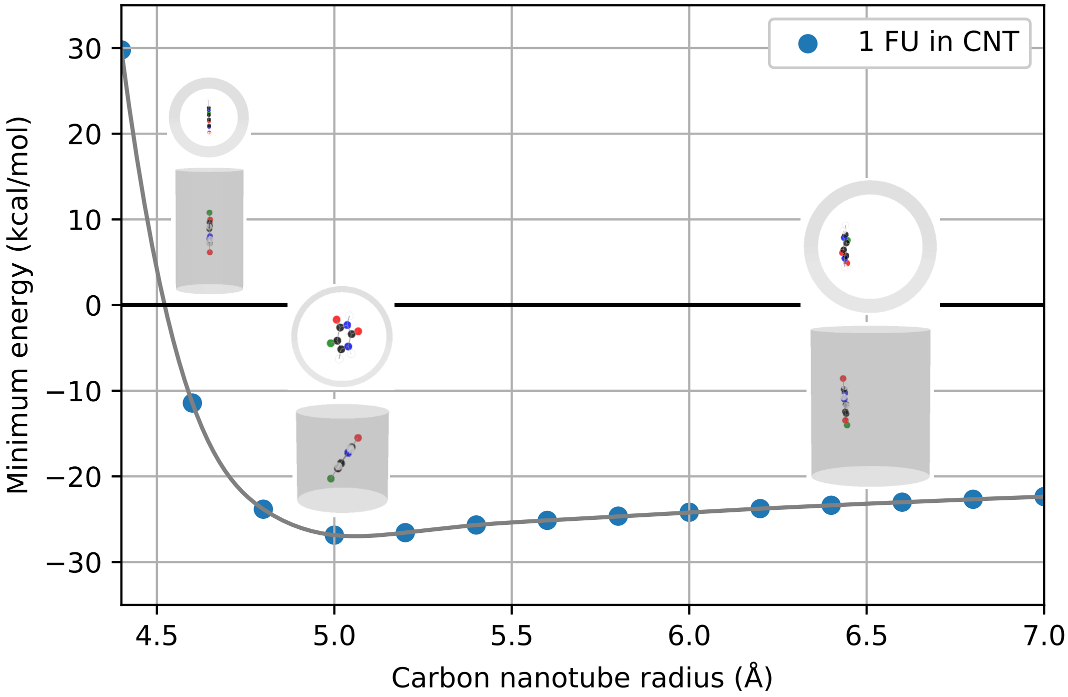


Figure S.6: Configurations of one fluorouracil encapsulated in CNT of various tube radii.

# 3 Equilibrium configuration of two drug molecules encapsulated in carbon nanotube

Figures S.7 to S.10 depict the equilibrium configurations for two encapsulated molecules, namely fluorouracils, proflavines, methylene blues, and doxorubicins, respectively. As shown in Table 1, each case exhibits two minimum energy states. Therefore, we present the configurations corresponding to both the global (two perspectives) and local (two perspectives) minima in these figures.

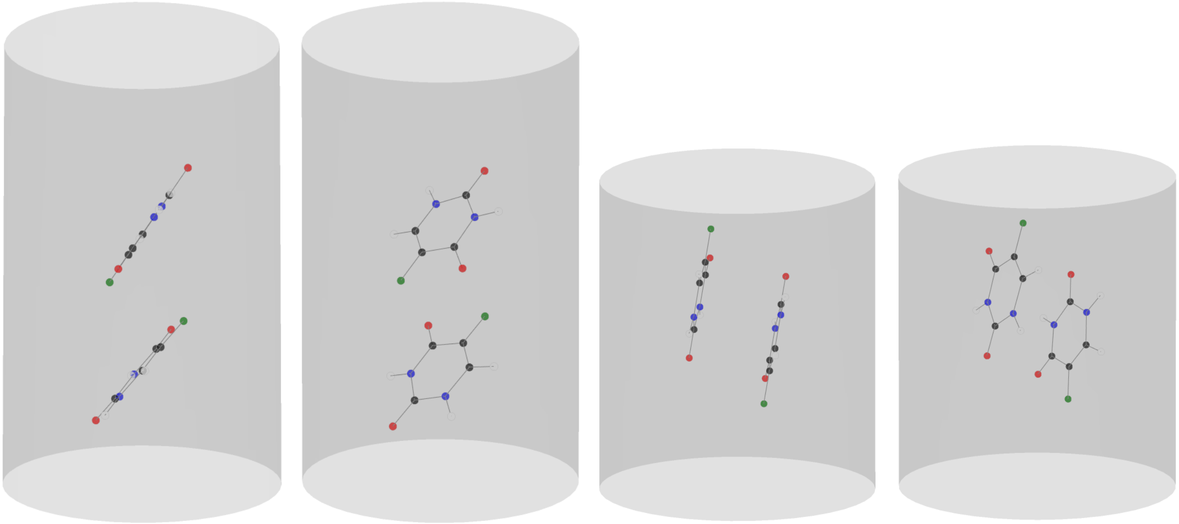

(a) (b) (c) (d)

Figure S.7: Two fluorouracils encapsulated in CNT (a), (b) at global minimum of tube radius 5.1391 Å, and (c), (d) at local minimum of tube radius 5.5009 Å.

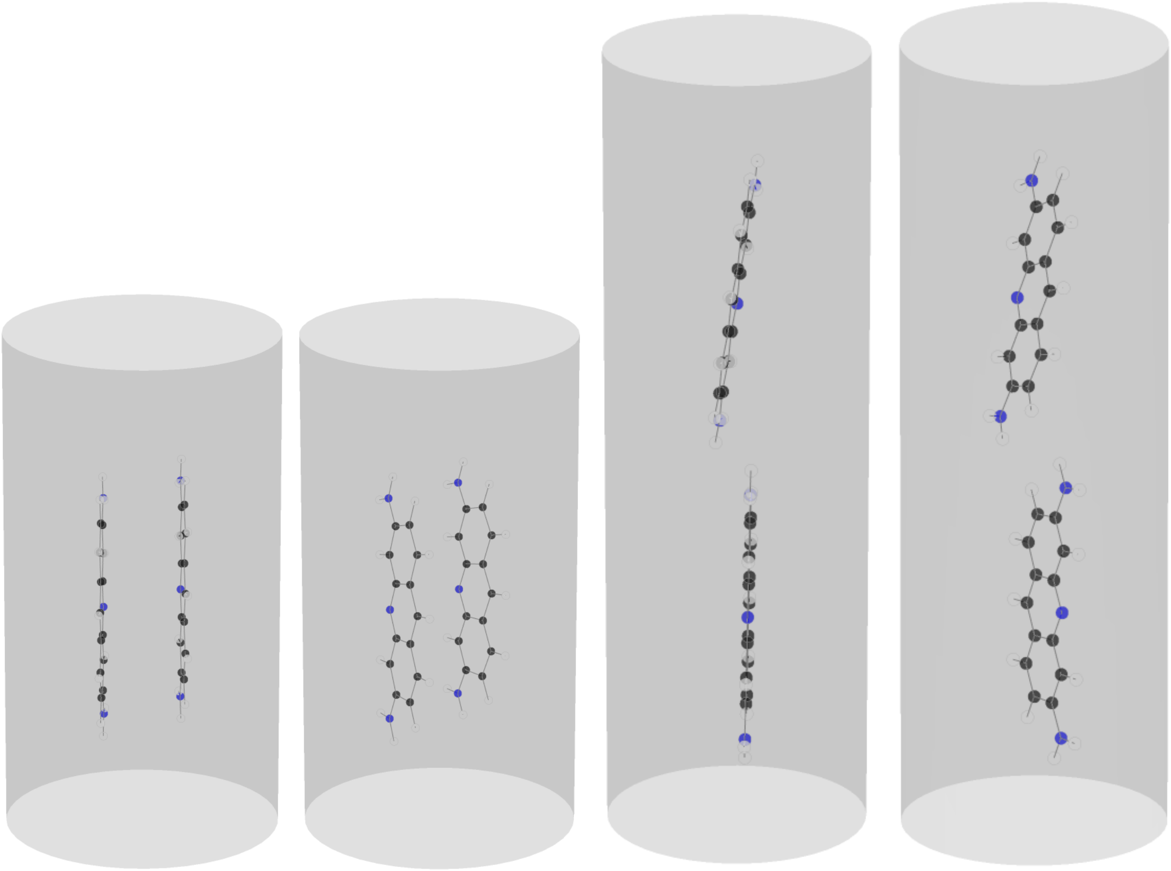

(a) (b) (c) (d)

Figure S.8: Two proflavines encapsulated in CNT (a), (b) at global minimum of tube radius 5.7604 Å, and (c), (d) at local minimum of tube radius 5.2054 Å.

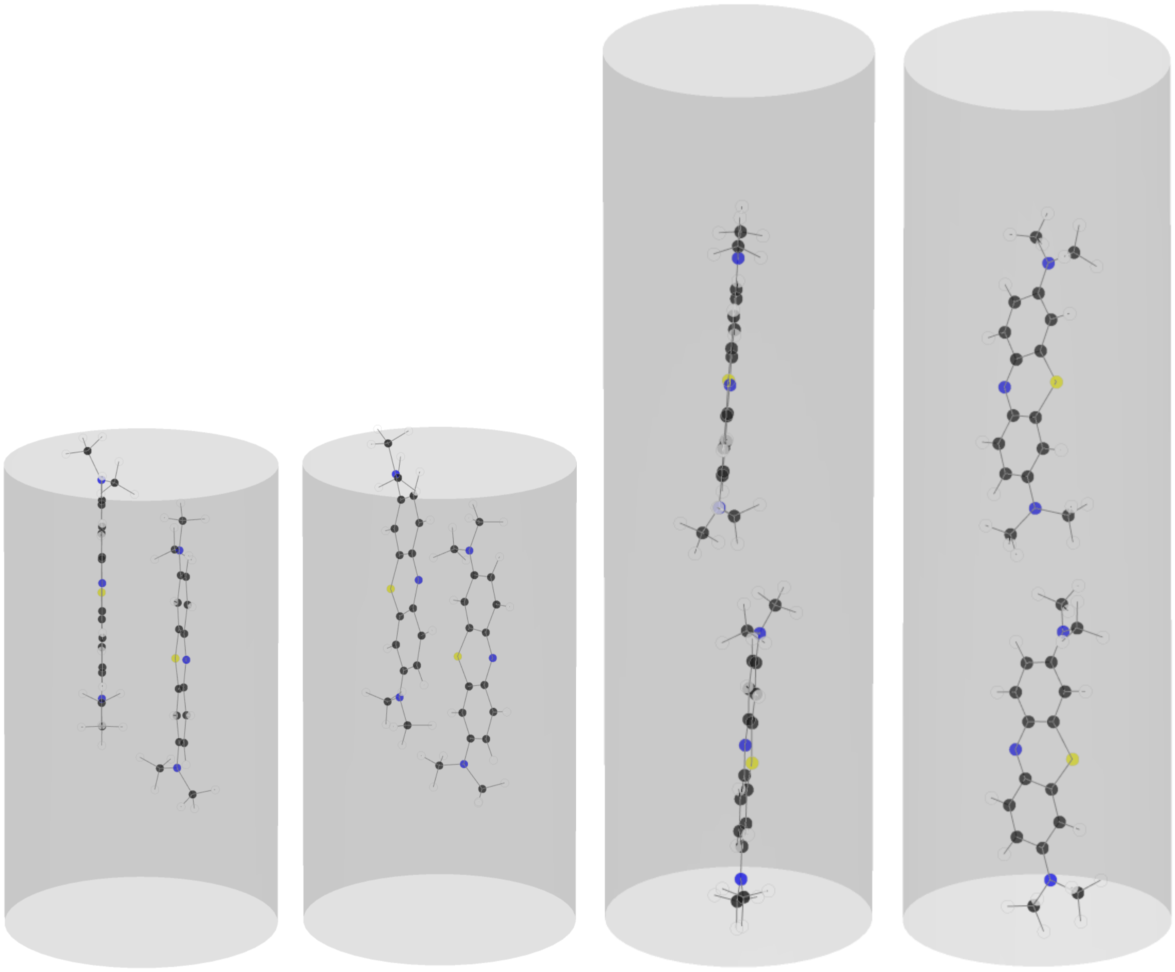

(a) (b) (c) (d)

Figure S.9: Two methylene blues encapsulated in CNT (a), (b) at global minimum of tube radius 6.0795 Å, and (c), (d) at local minimum of tube radius 5.4805 Å.

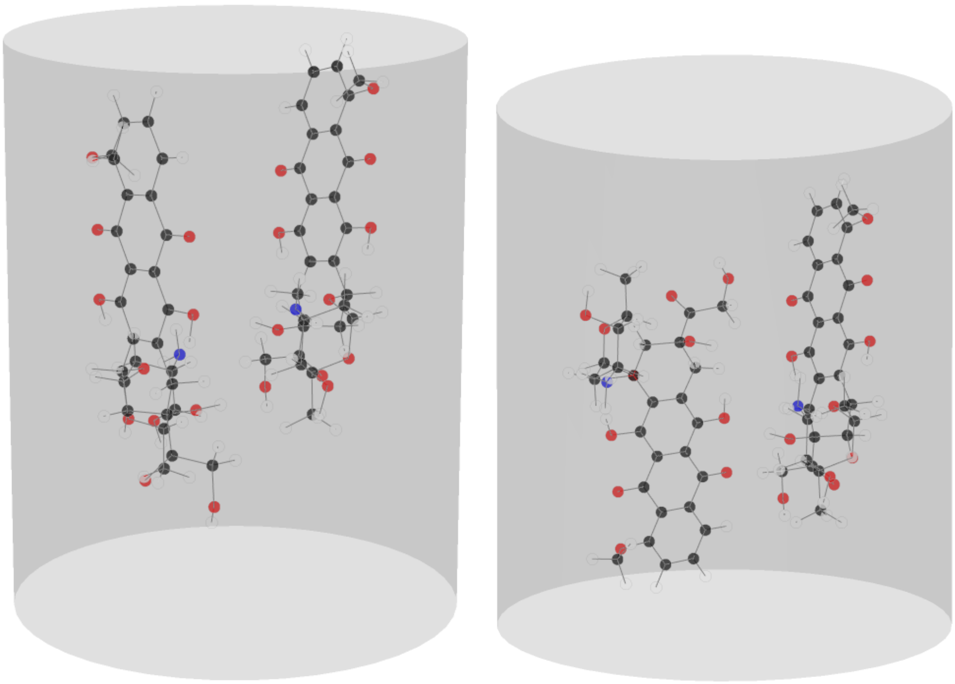

(a) (b)

Figure S.10: Two doxorubicins encapsulated in CNT (a) at global minimum of tube radius 8.1662 Å the carbon planes of drugs point in the same direction, and (b) at local minimum of tube radius 8.9970 Å the carbon planes of drugs points in the opposite direction.
